# Analysis of the Chloroplast Protein Kinase Stt7 during State Transitions

**DOI:** 10.1371/journal.pbio.1000045

**Published:** 2009-03-03

**Authors:** Sylvain Lemeille, Adrian Willig, Nathalie Depège-Fargeix, Christian Delessert, Roberto Bassi, Jean-David Rochaix

**Affiliations:** 1 Department of Molecular Biology University of Geneva, Geneva, Switzerland; 2 Department of Plant Biology, University of Geneva, Geneva, Switzerland; 3 University of Verona, Faculty of Sciences, Verona, Italy; Stanford University, United States of America

## Abstract

State transitions allow for the balancing of the light excitation energy between photosystem I and photosystem II and for optimal photosynthetic activity when photosynthetic organisms are subjected to changing light conditions. This process is regulated by the redox state of the plastoquinone pool through the Stt7/STN7 protein kinase required for phosphorylation of the light-harvesting complex LHCII and for the reversible displacement of the mobile LHCII between the photosystems. We show that Stt7 is associated with photosynthetic complexes including LHCII, photosystem I, and the cytochrome *b*_6_ *f* complex. Our data reveal that Stt7 acts in catalytic amounts. We also provide evidence that Stt7 contains a transmembrane region that separates its catalytic kinase domain on the stromal side from its N-terminal end in the thylakoid lumen with two conserved Cys that are critical for its activity and state transitions. On the basis of these data, we propose that the activity of Stt7 is regulated through its transmembrane domain and that a disulfide bond between the two lumen Cys is essential for its activity. The high-light–induced reduction of this bond may occur through a transthylakoid thiol–reducing pathway driven by the ferredoxin-thioredoxin system which is also required for cytochrome *b*_6_ *f* assembly and heme biogenesis.

## Author Summary

To grow optimally, photosynthetic organisms need to constantly adjust to changing light conditions. One of these adjustments, called state transitions, allows light energy to be redistributed between the two photosynthetic reaction center complexes in a cell's chloroplasts. These complexes act in concert with other components of the photosynthetic machinery to turn light energy into cellular energy. A key component in the regulation of state transitions is the chloroplast protein Stt7 (also known as STN7), which can modify other proteins by adding a phosphate group. When light levels change, the oxidation level of a pool of another chloroplast component, plastoquinone, changes, which in turn activates Stt7, inducing it to phosphorylate specific proteins of the light-harvesting complex of one reaction center. As a result, a portion of this light-harvesting complex is transferred from one photosynthetic reaction center to the other, thereby optimizing photosynthetic efficiency. Here, we have addressed the configuration of Stt7 within the thylakoid membrane of the chloroplast and the molecular mechanisms underlying its activation. Our data reveal that the level of Stt7 protein changes drastically under specific environmental conditions, that the protein does not need to be present in a one-to-one ratio with its targets for activity, and that it associates directly with a number of components of the photosynthetic machinery. The protein-modifying domain of Stt7 is exposed to the outer side of the thylakoid membrane, whereas the domain critical for regulation of its activity lies on the inner side of the thylakoid membrane. These results shed light on the molecular mechanisms that allow photosynthetic organisms to adjust to fluctuations in light levels.

## Abstract 3

The Stt7/STN7 chloroplast protein is involved in the phosphorylation and remodeling of the light-harvesting apparatus of photosynthetic organisms and plays a key role in the acclimation of the photosynthetic machinery following changes in light levels.

## Introduction

Photosynthetic organisms are constantly subjected to changes in light conditions. These organisms have developed different mechanisms to rapidly acclimate to this changing environment. At one extreme, when the absorbed light excitation energy vastly exceeds the assimilation capacity of the photosynthetic apparatus, these organisms need to protect themselves. Excess light energy is dissipated as heat through nonphotochemical quenching, which involves conformational changes in the light-harvesting system of photosystem II [1]. In contrast, under low light, photosynthetic organisms optimize the absorption capacity of their antenna systems. This is especially true when changes in light quality occur that lead to the preferential stimulation of either photosystem II (PSII) or photosystem I (PSI), which are linked through the photosynthetic electron transport chain. Under these conditions, balancing of the light excitation energy between the antenna systems of PSII and PSI occurs through a process called state transitions [2–4]. Upon preferential excitation of PSII, the plastoquinone pool is reduced, a process that favors binding of plastoquinol to the Qo site of the cytochrome *b*_6_ *f* complex and leads to the activation of a thylakoid protein kinase required for the phosphorylation of the light-harvesting system of PSII (LHCII) [5,6]. In the green alga Chlamydomonas reinhardtii, the LHCII protein set consists of Type I (Lhcbm3, Lhcbm4, Lhcbm6, Lhcbm8, and Lhcbm9), Type II (Lhcbm5), Type III (Lhcbm2 and Lhcbm7), and Type IV (Lhcbm1) proteins, and of CP26 and CP29 [7]. Because of their nearly identical sequences and size, several of these Lhcbm proteins cannot be distinguished by SDS-PAGE. Most of them fractionate into two major bands called P11/P13 (Type I) and P17 (Type III). CP29, Lhcbm5, P11, P13, and P17 are phosphorylated during a state 1 to state 2 transition [7–9]. Although CP29 and Lhcbm5 are mobile during state transitions, it is not yet clear which among the other LHCII proteins of C. reinhardtii are mobile [10,11]. The phosphorylation of LHCII is followed by a displacement of LHCII from PSII to PSI, thus increasing the size of the PSI antenna at the expense of the PSII antenna and rebalancing the excitation energy between both photosystems. Binding of the mobile LHCII to PSI requires the PsaH subunit [12]. This state corresponds to state 2. The process is reversible as preferential excitation of PSI leads to the dephosphorylation of LHCII by unknown phosphatases and its return to PSII (state 1).

State transitions can be induced, not only by changes in light conditions, but also through changes in cellular metabolism. Thus, in C. reinhardtii, the process can be triggered when the level of ATP is low or when the cells are grown in the dark under anaerobiosis. These conditions lead to the influx of reducing equivalents into the plastoquinone pool and to the activation of the LHCII kinase [13]. Moreover, transition from state 1 to state 2 in C. reinhardtii is associated with a switch from linear to cyclic electron transfer [14,15]. In this alga, the major role of state transitions appears to be ATP homeostasis. In land plants, the LHCII kinase can also be activated in the dark by the addition of sugar compounds [16]. Recent studies further confirm that state transitions are not limited to the balancing of excitation energy between the photosystems but that they also play a major role in the adjustment of the light reactions with carbon metabolism [17].

In C. reinhardtii, transition from state 1 to state 2 causes the displacement of 80% of LHCII from PSII to PSI, as deduced from the measurements of the quantum yield of PSI and PSII charge separation [18]. In contrast in land plants, only 15% of LHCII is mobile during state transitions, although this process is associated with considerable structural rearrangements of the thylakoid membranes [19]. The large size of the mobile LHCII antenna leads to significant changes in fluorescence yield during state transitions in C. reinhardtii, a feature that has been exploited for the screening of mutants deficient in this process [9,20]. Such a screen has revealed the existence of the thylakoid Ser-Thr protein kinase Stt7 (AA063768) [21]. Mutants deficient in this kinase are deficient in LHCII phosphorylation and fail to undergo a transition from state 1 to state 2. The Stt7 protein kinase is associated with the thylakoid membrane and contains a potential transmembrane domain upstream of the catalytic kinase domain. In Arabidopsis thaliana, the ortholog STN7 (NP_564946) is also specifically involved in LHCII phosphorylation and state transitions [21,22]. At this time, it is not yet clear whether Stt7 and STN7 act directly on LHCII or whether they act further upstream as part of a kinase cascade. The cytochrome *b*_6_ *f* complex plays a key role in the activation of the kinase [4]. It is thus very likely that Stt7/STN7 interacts directly with this complex. If LHCII is the substrate of Stt7/STN7, an interaction between the two is expected. Here, we have used coimmunoprecipitations and pull-down experiments to show that interactions of this type do indeed occur. Our data reveal that Stt7 acts in catalytic amounts. We also show that Stt7 contains a transmembrane region with the catalytic domain on the stromal side of the thylakoid membrane and the N-terminal region in the lumen. This domain appears to play a key role in the regulation of the kinase activity.

## Results

### Stt7 Is Associated with a Large Molecular Weight Complex

To understand how the Stt7 kinase functions, we first estimated its abundance, in particular its molar ratio compared with the cytochrome *b*_6_ *f* complex under state 2 conditions. An antibody was raised against Stt7 and the amount of Stt7 was estimated using recombinant Stt7 protein for calibration. A similar calibration was performed with the cytochrome *b*_6_ *f* complex (see Figure S1). This analysis revealed that the molar ratio between Stt7 and the cytochrome *b*_6_ *f* complex is 1:20, clearly indicating that Stt7 is present at substoichiometric levels compared to the photosynthetic complexes.

**Figure 1:**
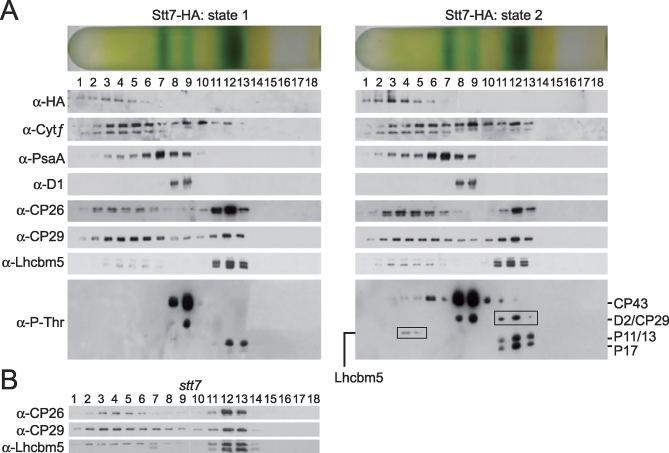
Fractionation of Stt7 and Chlorophyll-Protein Complexes by Sucrose Density Gradient Centrifugation (A) Thylakoid membranes from Stt7-HA cells in state 1 and state 2 were solubilized with n-dodecyl-β-maltoside, and the chlorophyll-protein complexes were separated by centrifugation on a sucrose density gradient. Proteins from the fractions of the gradients corresponding to Stt7-HA were separated by SDS-PAGE. Immunoblotting was performed with the indicated antibodies; α-P-Thr, anti–phospho-Thr. The identity of the phosphorylated proteins in the lower panel was determined by immunoblotting with antisera from the indicated proteins. In the immunoblot with a-P-Thr, signals corresponding to CP29 and Lhcbm5 are framed. (B) Immunoblots prepared as in (A) except that the thylakoid membranes from *stt7* were used. P11/13 and P17 are the major LHCII proteins of C. reinhardtii and correspond to Type I and Type III LHCII proteins, respectively.

The Stt7/STN7 protein kinase has been shown to be associated with the thylakoid membrane [21]. However, it is not known whether Stt7/STN7 acts singly or whether it is associated with other proteins in a larger complex whose composition might change during state transitions. To test this possibility, thylakoid membranes from the *stt7* mutant complemented with Stt7 containing a haemagglutinin (HA)-tag at its C-terminal end (Stt7-HA) were isolated. Membranes were prepared from cells in state 1 obtained by illumination in low light (6 μmol m^−2^ s^−1^) in the presence of DCMU (3-(3,4-dichlorophenyl)-1,1-dimethylurea) for 30 min and in state 2 by incubating the cells under anaerobic conditions for 30 min in the dark. The occurrence of state transitions was verified by measuring the change in maximum fluorescence (Fmax). The thylakoid membranes were solubilized with n-dodecyl-β-maltoside and fractionated by sucrose density gradient centrifugation. Individual fractions of the two gradients from Stt7-HA thylakoid membranes were separated by PAGE and then tested by immunoblot analysis using antibodies directed against HA, Cyt*f*, PsaA, D1, CP26, CP29, and Lhcbm5 (Figure 1A). Under both state 1 and state 2 conditions, the Stt7 protein kinase was associated with a large complex that partly overlaps with the high molecular weight fractions of PSI and the cytochrome *b*_6_ *f* complex but not with PSII (Figure 1A). No major changes in the distribution of the thylakoid complexes were observed under the state 1 and state 2 conditions used. A similar distribution of the complexes was found in the *stt7* mutant, confirming that the high molecular weight LHC complexes are not only formed under state 2, but also under state 1 conditions (Figure 1B).

Although the level of Stt7-HA was between 25%–50% compared to Stt7 in wild-type cells (Figure S2), state transitions proceeded to the same extent as in the wild type (unpublished data). Moreover, the amount of photosynthetic complexes was the same in Stt7-HA, *stt7*, and wild-type cells (Figure S2). As expected, immunoblots with an anti–P-Thr antiserum revealed increased phosphorylation of several proteins in state 2, notably the major LHCII proteins P11, P13, and P17 in the wild-type strain (Figure 1A). Moreover, a weak phosphorylation signal corresponding to Lhcbm5 was detected under state 2 conditions in the same high molecular weight fractions containing PSI. Although an increase of phosphorylation was also observed for the PSII core proteins CP43 and D2 under state 2 conditions, in other experiments, no significant increase of phosphorylation of these proteins was detected between state 1 and state 2.

### The Level of Stt7 Decreases under Prolonged State 1 Conditions

**Figure 2:**
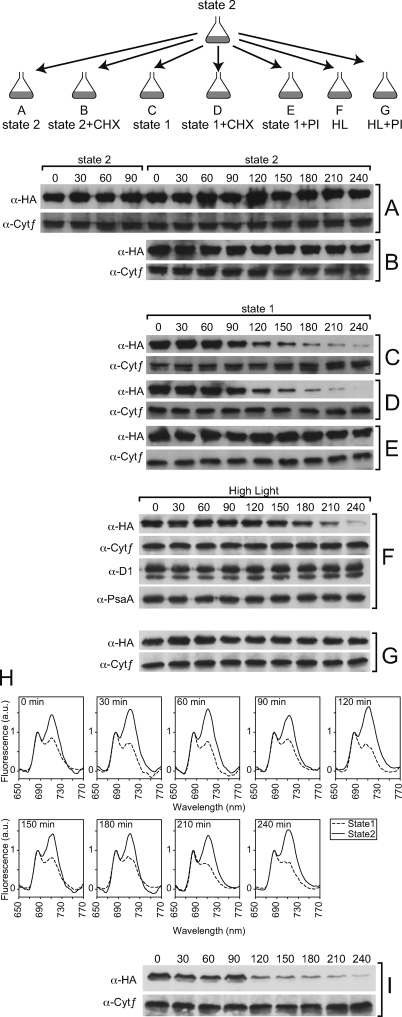
Levels of Stt7 Decrease under Prolonged State 1 Conditions and High Light Cells were grown for 120 min under state 2 conditions and subsequently maintained in state 2 (dark + anaerobiosis) or subjected to state 1 conditions (light + strong aeration) or to high light (900 μmol photons m^−2^ s^−1^) for 240 min. At various time intervals, total proteins were extracted and the amount of Stt7-HA was measured by immunoblotting with HA antiserum. Cyt*f* was used as a loading control. Top: outline of the experimental conditions (for details, see Materials and Methods). (A) Cells maintained under continuous state 2 conditions. (B) Same as (A), but with addition of cycloheximide after the initial culture was maintained in state 2 for 120 min. (C) Cells in state 2 were subjected to state 1 conditions after 120 min. (D) Same as (C), but with addition of cycloheximide after 120 min. (E) Same as (C) but with addition of protease inhibitors after 120 min. (F) Cells in state 2 were subjected to high light. The levels of PSII and PSI were assessed by immunoblotting with antibodies against D1 and PsaA, respectively. (G) Same as (F), but with addition of leupeptin. Total proteins were examined by immunoblotting with HA and Cyt*f* antibodies. (H) Cells shifted to state 1 conditions as shown in (C) were tested for transition from state 1 to state 2 at different times. In this case, transition to state 2 was induced by adding 5 μM FCCP and assayed after 15 min by measuring low-temperature fluorescence emission spectra for each time point. (I) Stt7 levels of the cells used in (H) were determined by immunoblotting with HA antiserum.

The immunoblots in Figure 1A indicate that the levels of Stt7 are significantly higher in state 2 than in state 1. To examine this further, cells containing Stt7-HA were grown for 2 h under state 2 conditions, and growth was continued for 4 h either under state 2 or state 1 conditions (Figure 2). At different time points, aliquots of cells were processed for immunoblot analysis with HA and Cyt*f* antibodies. Whereas the level of Stt7 remained the same under continuous state 2 conditions, its level decreased gradually 1 h after the shift to state 1 conditions. It decreased 4-fold after 2 h and more than 20-fold after 4 h (Figure 2C). After shifting the cells to state 2 conditions, the level of Stt7 increased 2-fold after 2 h but did not reach its initial value (unpublished data). Addition of cycloheximide did not affect the decline of Stt7, indicating that no newly synthesized protease is involved in this process (Figure 2D). However, addition of a protease inhibitor mixture to the cells abolished the degradation of Stt7 under state 1 conditions (Figure 2E).

The degradation of Stt7-HA under prolonged state 2 conditions monitored by immunoblotting with the HA antiserum could be due to the removal of the HA tag from Stt7. To test this possibility, we repeated this experiment with the Stt7-HA strain by using both Stt7 and HA antiserum. In both cases, a decline of the Stt7 kinase was confirmed under state 2 conditions (Figure S3A). We checked that this decrease also occurs with untagged Stt7 (Figure S3B). To identify the type of proteases involved, different protease inhibitors were tested under the same conditions as above: ACA (ɛ-aminocaproic acid) (Sigma) (50 mM), AEBSF (4-(2-aminoethyl)-benzenesulfonyl fluoride hydrochloride) (Roche) (5 mM), NEM (*N*-ethylmaleimide) (Sigma) (10 mM), phenylmethanesulfonyl fluoride (Sigma) (5mM), and EDTA (50 mM). Leupeptin and NEM were also used at different concentrations (see Figure S4). Samples were taken and analyzed at different time points. Whereas NEM completely prevented the breakdown of Stt7, the serine protease inhibitors ACA and AEBSF had no effect, and EDTA enhanced this process (Figure S4A and S4B). Other inhibitors of cysteine proteases besides NEM, such as E64 and leupeptin, prevented Stt7 degradation under prolonged state 1 conditions (Figure S4C, S4D, and S4E). Although no convincing proof of chloroplast Cys proteases has been reported, their existence cannot be excluded. A bioinformatic search for Cys proteases in plastids of A. thaliana did not reveal any convincing candidate (see Text S1).

As high-light treatment is known to lead to the inactivation of the LHCII protein kinase [23,24], we tested whether Stt7 was stable under these conditions. Cells adapted to state 2 were subjected to high-light treatment (900 μmol m^−2^ s^−1^), and the Stt7 levels were determined by immunoblotting at different times. Under these conditions, a steady decrease of Stt7 was observed (Figure 2F), which could be fully prevented by addition of leupeptin (Figure 2G). Under the same light regime, the level of PSII and of PSI were nearly unaffected (Figure 2F). However, measurements of the ratio of variable (Fv) over maximal florescence (Fv/Fmax) revealed that this ratio decreased from 0.7 to 0.2, indicating photodamage to PSII without apparent decrease of D1 protein level.

### Stt7 Acts in Catalytic Amounts

We took advantage of the decrease in Stt7 under prolonged state 1 conditions to test whether the cells are still able to switch from state 1 to state 2 under these conditions. Cells were first grown under state 1 conditions as shown in Figure 2. After different time periods, cells were collected and assayed for state transitions using the uncoupler FCCP (carbonyl cyanide p-fluoromethoxyphenylhydrazone), a known inducer of transition to state 2 [13]. The fluorescence emission spectra at low temperature and the level of Stt7 were measured under state 1 and state 2 conditions. Figure 2H shows that transition to state 2 occurred readily in these cells collected after 4 h under state 1 conditions, although the amount of Stt7 protein kinase was decreased 20-fold (Figure 2I). This indicates that the Stt7 kinase acts in catalytic amounts.

### Stt7 Interacts with the Cytochrome *b*_6_ *f* Complex, LHCII, and PSI

**Figure 3:**
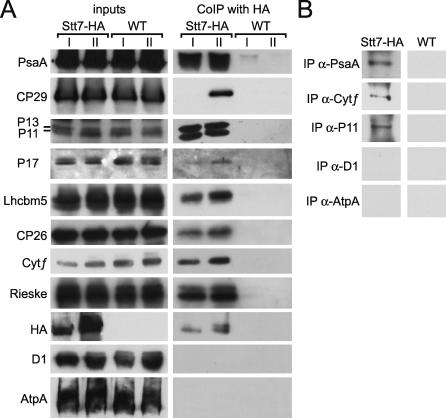
Coimmunoprecipitations of Stt7 with LHCII, the Cytochrome *b*_6_ *f* Complex, and PSI (A) Right panel: thylakoid membranes from the *stt7* mutant strain complemented with Stt7-HA and wild-type (WT) cells in state 1 and state 2 were solubilized with n-dodecyl-β-maltoside, and proteins were coimmunoprecipitated (CoIP) with HA antibodies. The immunoprecipitates were separated by SDS-PAGE and immunoblotted with the antibodies indicated. Left panel: the inputs are shown and represent 10% of the samples used for the coimmunoprecipitations. (B) Thylakoid membranes from the Stt7-HA or wild-type (WT) strain were processed as above and coimmunoprecipitated with PsaA, Cyt*f*, D1, and AtpA antibodies. The immunoprecipitates were separated by SDS-PAGE and immunoblotted with HA antibodies.

Activation of the LHCII kinase depends critically on the cytochrome *b*_6_ *f* complex [4]. Moreover, this kinase is likely to interact with its putative substrate LHCII. To test whether the Stt7 kinase interacts with the cytochrome *b*_6_ *f* complex and/or LHCII, thylakoid membranes from the Stt7-HA strain in state 1 or state 2 were solubilized with dodecyl maltoside and immunoprecipitated with HA antiserum. The immunoprecipitates were fractionated by SDS-PAGE and immunoblotted with antibodies against several known thylakoid proteins. Figure 3A shows that the LHCII proteins P13, P11, P17, CP29, CP26, and Lhcbm5 were coimmunoprecipitated with the Stt7 kinase. The signal obtained with CP29 was only detectable under state 2 conditions. Signals were also observed with Cyt*f* and Rieske protein as well as with the PSI subunit PsaA. In contrast, no interaction was observed between Stt7 and D1 from PSII and with subunit α of ATP synthase (Figure 3A), indicating that the interactions detected between Stt7-HA and the other photosynthetic proteins are specific. Furthermore, no signal was observed with the untagged wild-type strain (Figure 3A). Reciprocal immunoprecipitations with PsaA, Cyt*f*, and P11 antibodies confirmed the interaction of these proteins with Stt7 (Figure 3B).

**Figure 4:**
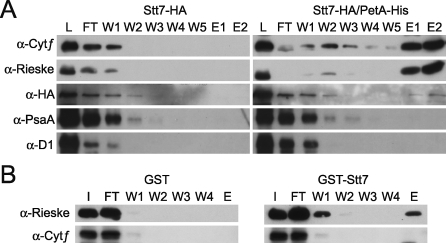
Stt7 Cofractionates with the Cytochrome *b*_6_ *f* Complex (A) Thylakoid membranes from strains containing Stt7-HA and Stt7-HA with His-tagged Cyt*f* (PetA-His) were solubilized with n-dodecyl-β-maltoside and fractionated by Ni-NTA chromatography. E1, E2 eluates (300 mM imidazole) were analyzed by PAGE and immunoblotting with the indicated antibodies. FT, flow-through; L, loading (10 mM imidazole); W1–W5, washes (20 mM imidazole). (B) Stt7 interacts with the Rieske protein. GST and GST-Stt7 fusion protein were incubated separately with purified solubilized cytochrome *b*_6_ *f* complex from wild-type cells. The eluates (E) (100 mM DTT, 2% SDS) were analyzed by PAGE and immunoblotting with Rieske and Cyt*f* antibodies. FT, flow-through; I, input; W1–W4, washes (PBS).

To further test the interaction of Stt7 with the cytochrome *b*_6_ *f* complex, the Stt7-HA strain was transformed with *petA* containing a His tag at its 3′-end [25]. After testing the homoplasmic state of the transformed strain for *petA-His*, thylakoid membranes were isolated, solubilized, and the cytochrome *b*_6_ *f* complex was purified on a Ni-NTA column. After washing the column, immunoblotting of the eluted fraction revealed that a small portion of Stt7 kinase was associated with the tagged cytochrome *b*_6_ *f* complex, but not with the untagged strain (Figure 4A). To determine which of the subunits of the cytochrome *b*_6_ *f* complex interacts with Stt7, a pull-down assay with GST-Stt7 and solubilized purified cytochrome *b*_6_ *f* complex was performed. The results (Figure 4B) show that the Rieske protein, but not Cyt*f*, could be eluted from the GST-Stt7 column, indicating that Stt7 interacts with the Rieske protein.

### Orientation of the Stt7 Kinase in the Thylakoid Membrane

**Figure 5:**
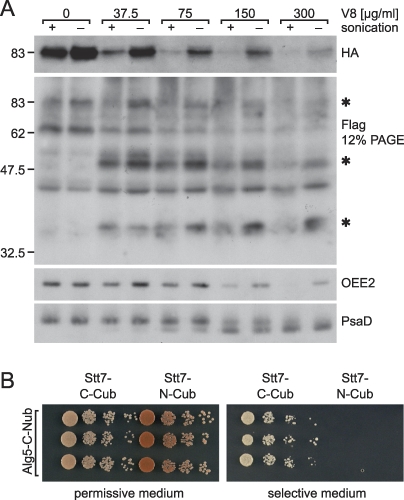
Stt7 Contains a Transmembrane Region with Its N-Terminal End in the Thylakoid Lumen (A) Thylakoid membranes from a strain containing FLAG-Stt7-HA were subjected to increasing concentrations of V8 protease at room temperature, subjected to PAGE, and immunoblotted with HA, FLAG, PsaD, and OEE2 antibodies. An asterisk (*) indicates degradation products of Stt7 revealed with the FLAG antiserum. (B) Split-ubiquitin assays were performed with fusions of the C-terminal half (Cub) of ubiquitin with either the N- or C-terminal end of Stt7 and the ER protein Alg5 fused at its C-terminal end with the N-terminal half of ubiquitin (Nub). Yeast colonies were plated on permissive (-L-W) and selective (-Ade-H-L-W) media with 10-fold and two 5-fold serial dilutions, respectively.

Although a putative transmembrane domain within Stt7 is predicted by several algorithms [21], the presence of four Pro residues within this domain raises some questions, and two models need to be considered. In the first, the N-terminal end of Stt7 would be separated from the large catalytic domain of Stt7 by a transmembrane domain. In the second model, Stt7 could be localized entirely on one side of the thylakoid membrane. To distinguish between these possibilities, the Stt7 protein was tagged with FLAG and HA at its N-and C-terminal ends, respectively. It should be noted that FLAG-Stt7-HA is not functional but that it stably accumulates in the thylakoid membranes (see below). Because the presumed substrates of the Stt7 kinase are localized on the stromal side of the thylakoid membrane, it is expected that the kinase domain of Stt7 is also located on the stromal side. This was tested by isolating intact thylakoid membranes from the strains containing Stt7-HA or FLAG-Stt7-HA and by subjecting the thylakoid membranes to mild digestion with protease V8. The resulting protein extracts were then examined by PAGE and immunoblotting using antibodies directed against HA, FLAG, PsaD, and OEE2. PsaD is known to be partially exposed to the stromal side, whereas OEE2 is entirely located on the lumenal side of the thylakoid membrane. OEE2 and the main body of PsaD are thus expected to be protected from any external protease. Under conditions (75 μg/ml V8) in which proteolysis mildly affected the OEE2 protein and led to partial digestion of PsaD, the level of Stt7 was significantly decreased as measured with HA antibodies, confirming that the kinase domain is located on the stromal side of the membrane (Figure 5A). Similar results were obtained with Stt7-HA (unpublished data). In contrast when antibodies against FLAG were used, products of smaller size were detected, indicating that the N-terminal end of Stt7 is localized on the lumenal side of the thylakoid membrane (Figure 5A). Sonication of the thylakoid membranes followed by V8 protease treatment revealed that the levels of protected fragments detected with the FLAG antibodies were significantly reduced as also observed with the lumenal protein OEE2 (Figure 5A). Sonication also led to enhanced degradation of the HA-tag of Stt7, presumably because the domain of the kinase exposed to the stroma was more accessible to the protease under these conditions and/or the thylakoid membrane was damaged. In contrast, no difference was observed for PsaD with and without sonication.

The orientation of Stt7 was further tested with the yeast split-ubiquitin system [26]. The C-terminal fragment of ubiquitin (Cub) was fused to either the N- or C-terminal end of Stt7 and expressed in yeast together with the N-terminal end of ubiquitin (Nub) fused to the C-terminal end of the endoplasmic reticulum (ER) protein Alg5, which places Nub on the cytoplasmic side of the membrane. Ubiquitin was reconstituted only with the construct in which Cub was fused to the C-terminal end of Stt7 but not when it was fused to the N-terminal end (Figure 5B). Because membrane proteins usually insert with their lumen domain in the periplasmic space of yeast, the two-hybrid results are fully compatible with the topology of Stt7 derived from the protease protection studies.

### Mutations in the N-Terminal Region of Stt7 Abolish Kinase Activity

**Figure 6:**
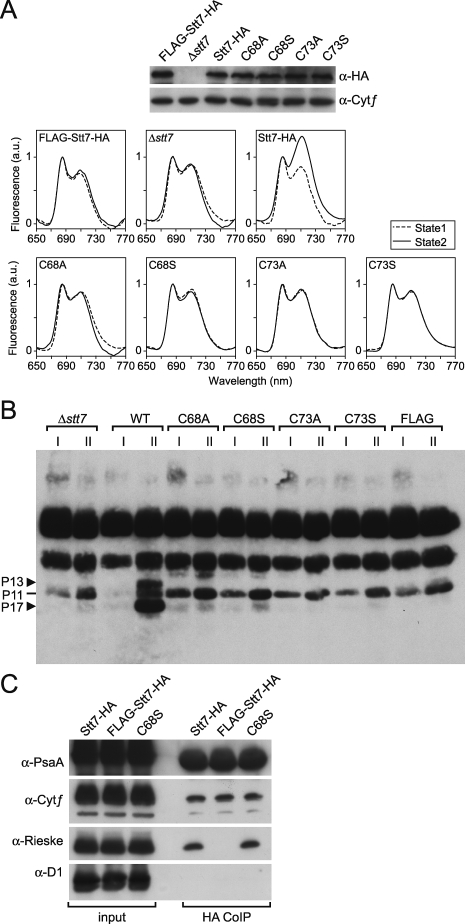
Analysis of Mutants of Stt7 Affected in the N-Terminal Region Transformants of *stt7* containing mutant forms of Stt7-HA with changes of Cys68 and Cys73 to Ala/Ser or with a FLAG-tag (FLAG-Stt7-HA) at the N-terminal end were used. (A) Cys68 and Cys73 of Stt7 are essential for state transitions and LHCII phosphorylation. Fluorescence emission spectra at 70 K of wild type, *stt7*, and the different transformants under state 1 and state 2 conditions. Right: immunoblots of total extracts from these strains and Stt7-HA with HA antibodies. (B) Cys68 and Cys73 are required for LHCII phosphorylation under state 2 conditions. Membrane proteins from the wild type and the indicated transformants under state 1 and state 2 conditions were fractionated by SDS PAGE and immunoblotted with an anti–P-Thr antiserum. The bands missing in the transformants are indicated by two arrows. (C) Insertion of a FLAG tag at the N-terminal end of Stt7 specifically prevents its interaction with the Rieske protein. Thylakoids from Stt7-HA, FLAG-Stt7-HA, and Stt7-C68S were solubilized with n-dodecyl-β-maltoside and immunoprecipitated with HA antiserum. The immunoprecipitate was fractionated by PAGE and immunoblotted with the indicated antibodies.

The transmembrane domain of Stt7 near its N-terminal end is preceded by a region that contains two conserved Cys separated by four residues that are also conserved in the orthologous STN7 kinase of *Arabidopsis* [21]. In land plants, it has been shown that under high light, the LHCII kinase is inactivated through the ferredoxin-thioredoxin system [23,24]. One possibility is that these conserved Cys are targets of this redox system. To test the role of these residues, the two Cys were changed individually to Ser or Ala by transforming the *stt7* mutant with the Stt7-HA-C68S/A and Stt7-HA-C73S/A constructs. In all four cases, the mutant kinase accumulated as in Stt7-HA cells (Figure 6A). However, the low-temperature fluorescence emission spectra measured under conditions inducing state 1 or state 2 were nearly identical in these mutants, indicating that they are deficient in state transitions (Figure 6A). As expected, an increase of PSI fluorescence at 715 nm, which is characteristic for state 2, was detected in the rescued Stt7-HA strain. Moreover, the Cys mutants failed to phosphorylate LHCII under state 2 conditions (Figure 6B). Thus, the Cys residues are critical for kinase activity, although they are separated from the catalytic kinase domain by the transmembrane region.

The insertion of a FLAG-tag near the N-terminal end of mature Stt7 abolished state transitions and kinase activity but did not affect the stable accumulation of the protein (Figure 6A and 6B). Moreover, the presence of the FLAG tag specifically prevented the coimmunoprecipitation of Stt7 with the Rieske protein, but not with Cyt*f* (Figure 6C). At first view, this appears to contradict the results of the pull-down experiment, which indicate that the Rieske protein, but not Cyt*f*, interacts with Stt7. In the case of the pull-down experiment, recombinant Stt7 protein was used in which the transmembrane may not be correctly folded. This domain could be responsible for the binding to Cyt*f*. In the case of the immunoprecipitation, solubilized thylakoid membranes were used in which the interaction between Stt7 and Cyt*f* is preserved. The addition of the FLAG epitope appears to prevent proper interaction of Stt7 with the Rieske protein. In contrast, coimmunoprecipitations of Stt7-C68S occurred both with Cyt*f* and the Rieske protein (Figure 6C). Taken together, these results indicate that the N-terminal end of Stt7 plays a crucial role in the activation of its kinase activity and that this domain may be involved in the interaction with the Rieske protein.

## Discussion

### The Stt7 Protein Kinase Is Associated with a Large Molecular Weight Complex

State transitions lead to a considerable reorganization of the antenna systems of PSII and PSI in C. reinhardtii. Moreover, they are accompanied by large changes in the PSI-LHCI supercomplex [11,27]. Fractionation of solubilized thylakoid membranes by sucrose density gradient centrifugation revealed no major changes in the distribution of Stt7 and the LHCII proteins between PSII and PSI under state 1 and state 2 conditions. Interestingly, Stt7 is associated with a large molecular weight complex which cofractionates with the high molecular weight fractions of the cytochrome *b*_6_ *f* complex and PSI, but clearly not with PSII (Figure 1). These partial cofractionations of Stt7 with the cytochrome *b*_6_ *f* complex and PSI are compatible with the coimmunoprecipitation experiments (Figure 3). The exact composition of these complexes remains to be determined.

Our results on the fractionation of the complexes differ slightly from those of Takahashi et al. [11]. Whereas these authors found a clear shift of CP26, CP29, and Lhcbm5 towards the PSI region upon a transition from state 1 to state 2, which they attribute to the formation of a large PSI-LHCII supercomplex, we only detected a small increase in the ratio between high and low molecular weight fractions of the sucrose gradient in state 2 for CP26 and Lhcbm5 (Figure 1A). In the case of CP29, there was no significant difference in its distribution in state 1 and state 2. These differences may be due to the fact that state 2 and state 1 were induced through different means in our study and that of Takahashi et al. They used FCCP plus NaF and DCMU plus staurosporine for inducing state 2 and state 1, respectively, whereas we used anaerobiosis and vigorous aeration in the presence of DCMU. We further confirmed the presence of the LHCII proteins detected in the high molecular weight fractions under state 1 conditions in the *stt7* mutant.

Although we could not detect major changes in the distribution of the LHC complexes in the sucrose density gradient under state 1 and state 2 conditions, there were changes in the phosphorylation patterns. A phosphorylated form of Lhcbm5 was detectable in the high molecular weight fractions under state 2, but not state 1 conditions (Figure 1A). Takahashi et al. [11] observed phosphorylated forms of CP29 and Lhcbm5 in this fraction. A phosphorylated form of CP29 in the high molecular weight fraction was also reported by Kargul et al. [10]. The differences between these studies might be partly accounted for by the different specificities of the anti–P-Thr antibodies used.

### Stt7 Is Less Abundant under State 1 Conditions

A striking feature is that the level of Stt7 is significantly lower in state 1 than in state 2. This was further investigated by a state 2–state 1 time course experiment (Figure 2). Within 2 h after shifting from state 2 to state 1 conditions, the level of Stt7 decreased to one fourth of state 2 levels. The level of Stt7 decreased further to 2%–5% of state 2 levels after 4 h in state 1, indicating that Stt7 is unstable under prolonged state 1 conditions. Thus, Stt7 is more abundant and stable under conditions where it is active. The decrease of Stt7 under state 1 conditions could be prevented by addition of inhibitors of Cys proteases to intact cells. It is therefore possible that under state 2 conditions, Stt7 is more protected from proteases either because of a posttranslational modification, e.g., phosphorylation, or of its association with other proteins. Although experimental evidence for the presence of cysteine proteases in chloroplasts is weak, their existence and role in Stt7 turnover cannot be excluded. It is possible that other proteases are involved in this process, such as the FtsH and Deg proteases, which are known to degrade thylakoid membrane proteins [28]. Some Deg proteases are indeed sensitive to NEM [29].

We note that the decrease in Stt7 occurs only after a prolonged period in state 1, whereas state transition is a short-term acclimation response that occurs within minutes. It is therefore possible that the control of the Stt7 level is part of a long-term acclimation response. There is indeed evidence for a role of the ortholog STN7 of A. thaliana in such a process [30].

### The Level of Stt7 Is Decreased under High Light

The level of Stt7 decreases under high light. Under the same conditions, PSII levels remained constant. In land plants, the LHCII kinase is known to be inactivated through the ferredoxin-thioredoxin system under high light [31]. It is thus conceivable that the kinase is less stable when it is maintained for a prolonged period in its inactive state, induced either by state 1 conditions or high light. Although the redox state of the plastoquinone pool is critical for activation of the kinase, it does not solely determine the level of Stt7. Under state 1 conditions, the plastoquinone pool is oxidized, whereas it is expected to be reduced under high light.

### Stt7 Interacts with the cytochrome *b*_6_ *f* Complex, LHCII, and PSI

A close interaction between Stt7 and the cytochrome *b*_6_ *f* complex is apparent from the coimmunoprecipitation results. This complex is known to play a critical role for the activation of the kinase during a state 1 to state 2 transition [32]. Mutants deficient in cytochrome *b*_6_ *f* complex fail to phosphorylate LHCII and are blocked in state 1 [32].

The original state transition model postulates that upon activation of the Stt7 kinase through the cytochrome *b*_6_ *f* complex, the kinase is released from the complex to phosphorylate LHCII. However, we find that the association of the Stt7 kinase with the cytochrome *b*_6_ *f* complex does not markedly change between state 1 and state 2. One possibility is that another downstream kinase is phosphorylated by Stt7, which in turn phosphorylates LHCII. Alternatively, the Stt7 kinase may be part of a large supercomplex that includes the cytochrome *b*_6_ *f* complex and the PSII-LHCII complex. We have not been able to detect complexes of this kind, although it is possible that they are only formed transiently. The PetO subunit of the *C. reinhardtii b*_6_ *f* complex is known to be phosphorylated during state transitions [33]. Despite several attempts, we were unable to detect any interaction between Stt7 and PetO based on coimmunoprecipitations or yeast two-hybrid screens. It remains to be seen whether the phosphorylation of PetO depends on Stt7.

To identify which subunit of the cytochrome *b*_6_ *f* complex interacts with Stt7, pull-down experiments were performed. The Rieske protein was identified as an interactant (Figure 4B). Based on structural studies of the mitochondrial *bc*1 and of the chloroplast *b*_6_ *f* complexes, electron transfer between plastoquinol at the Qo site and cytochrome *f* (Cyt*f*) is mediated by the Rieske protein which moves from a proximal position when the Qo site is occupied by plastoquinol to a distal position when the Qo site is unoccupied [25,34,35]. It has been suggested that this dynamic behavior of the Rieske protein could be coupled to the activation of the Stt7 kinase [36–38]. Such a dynamic model is compatible with the low abundance of the Stt7 kinase with a molar ratio of 1:20 relative to the cytochrome *b*_6_ *f* complex found in this study. Assuming one cytochrome *b*_6_ *f* complex for one PSII core complex and an average of ten LHCII proteins per PSII reaction center [39], the molar ratio of Stt7 kinase to LHCII protein can be estimated at 1:200. At first sight, the kinase would have to phosphorylate several LHCII substrates and may undergo multiple rounds of activation. It is possible that the Stt7 kinase acts first on the LHCII located in the edges of the grana and that this phosphorylation induces extensive remodeling of the thylakoid membrane as reported recently during state transitions in A. thaliana [19]. These changes may facilitate the access of Stt7 to LHCII in the grana core. Alternatively, the observed strong LHCII phosphorylation under state 2 conditions could be due to very flexible movements of PSII-LHCII supercomplexes in the grana core and grana margins.

An intriguing component of the cytochrome *b*_6_ *f* complex is its single chlorophyll *a* molecule whose chlorine ring lies between helices F and G of the PetD subunit, whereas the phytyl chain protrudes near the Qo site [25]. Interestingly, mutants affected in the binding site of chlorophyll *a*, besides having reduced cytochrome *b*_6_ *f* turnover, also display a decreased rate of transition from state 1 to state 2 [36]. This region may thus either be an interaction site for the N-terminal region of Stt7 or it could act as a sensor for the presence of plastoquinol at the Qo site and initiate a signaling pathway through the chlorophyll *a* molecule towards the catalytic domain of Stt7 on the stromal side of the thylakoid membrane.

The coimmunoprecipitation experiments indicate that Stt7 is associated with LHCII in most cases under both state 1 and state 2 conditions. LHCII could be the direct substrate of Stt7 or, alternatively, Stt7 could be part of a multikinase complex, which ultimately phosphorylates LHCII. The only marked difference in coimmunoprecipitation between state 1 and state 2 was observed for CP29 (Figure 3). CP29 is particularly interesting. First, this monomeric LHCII together with CP26 and Lhcbm5 has been proposed to act as linker between the LHCII trimers and the dimeric PSII reaction center for the transfer of excitation energy [11,39–41]. Second, during transition from state 1 to state 2, CP29 undergoes hyperphosphorylation: in addition to Thr6 and Thr32, which are phosphorylated in state 1, Thr16 and Ser102 are phosphorylated in state 2 [10,42]. Third, electron microscopy (EM) analysis of PSII-LHCII complexes lacking CP29 could not distinguish between C2S2 and PSII monomeric complexes [39]. Fourth, in maize bundle sheath cells, which carry out mostly cyclic electron flow, the few remaining PSII complexes are monomeric [43]. Taken together, these results raise the possibility that the phosphorylation of CP29 in state 2 may act as a switch for cyclic electron flow with the detachment of CP29 from PSII and its monomerization.

The coimmunoprecipitation experiments also reveal an interaction of Stt7 with the PSI complex. This finding is surprising, as one would expect that the kinase acts on LHCII bound to PSII and that it would not interact with PSI. However, movement of PSI-LHCI complexes from the stromal lamellae to the grana margins following LHCII phosphorylation occurs in land plants, and it was proposed that the PSI absorption cross section is increased in this region through interaction of PSI-LHCI with the phosphorylated LHCII originating from the grana [44]. It is possible that the grana margins constitute a platform where the observed interactions of Stt7 with PSI could occur. It is not known whether the kinase is active or inactive when it is bound to PSI, and the role of this association remains to be determined.

### The N-Terminal Part of Stt7 Is Localized in the Thylakoid Lumen and Is Essential for Kinase Activity

**Figure 7:**
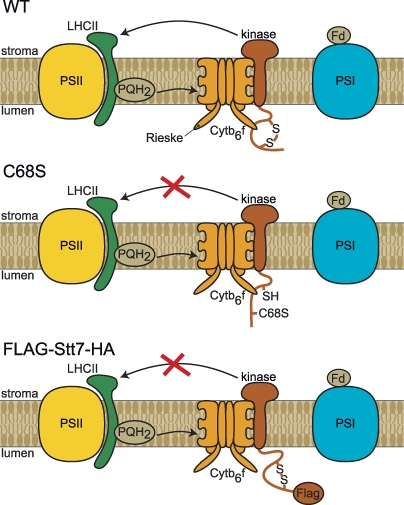
Model of Stt7 Kinase within the Thylakoid Membrane in Wild Type, Stt7-C68S, and FLAG-Stt7-HA The N-terminal part of Stt7 interacts with the lumenal domain of the Rieske protein. A disulfide bridge between Cys68 and Cys73 may be required for the activity of Stt7 that is abolished in Stt7-C68S. Interaction of Stt7 with Rieske protein is prevented by the insertion of a FLAG-tag at the N-terminal end of Stt7. The catalytic domain of Stt7 is on the stromal side of the thylakoid membrane where it could phosphorylate LHCII. The HA-tag of the Stt7-HA is at is C-terminal end on the stromal side of the thylakoid membrane. Fd, ferredoxin; PQH_2_, plastoquinol.

Analysis of the Stt7 amino acid sequence by bioinformatic means predicts the presence of a single transmembrane domain. However, this putative transmembrane domain contains four Pro residues that may prevent the formation of an α helix. It was therefore important to test the topology of Stt7 by experimental means using Stt7 tagged at its N-terminal end with FLAG and at its C-terminal end with HA. Using intact thylakoid membranes, we showed that whereas the C-terminal end of Stt7 is susceptible to protease digestion, the N-terminal end is protected, indicating that Stt7 indeed contains a transmembrane domain with the kinase domain on the stromal side and the N-terminal end in the lumen. The N-terminal region contains Cys68 and Cys73, which are conserved in the ortholog of Stt7 in land plants [21]. Among the seven Cys residues in Stt7, these are the only conserved Cys residues between Stt7 and STN7. In land plants, the LHCII kinase is inactivated by high-light treatment through the ferredoxin-thioredoxin system [31]. The two conserved Cys could therefore be the targets of this redox system and/or play a major role in the activation of the kinase. By changing either of the two Cys to Ala or Ser, the kinase was inactivated. There was no phosphorylation of LHCII under state 2 conditions, and the mutants were blocked in state 1. Possibly a disulfide bridge between these two Cys is required for kinase activity, or redox changes of this disulfide bridge are critical for its activity (Figure 7). The question arises how the redox state of these two Cys in the lumen is regulated through the redox state of the stromal compartment. Recently, at least two components of a transthylakoid thiol–reducing pathway have been identified in chloroplasts. The CcdA thiol disulfide transporter is a polytopic thylakoid protein with two highly conserved Cys in membrane domains, which is able to convey reducing power from the stroma to the lumen [45]. The second component is the Hcf164 thioredoxin-like protein, which acts as a thiol disulfide oxidoreductase [46,47]. This system, which is also conserved in bacteria, is thought to be involved in cytochrome *c* and cytochrome *b*_6_ *f* assembly but could also have additional roles. In this respect, given the tight physical association of Stt7 with the cytochrome *b*_6_ *f* complex and the requirement of this active complex for the activation of the kinase, it is tempting to propose that the redox state of the Cys68 and Cys73 couple is controlled through the same thioreduction system that operates in cytochrome *b*_6_ *f* assembly. These changes in redox state of Stt7 could in turn induce conformational changes of the kinase and affect both its activity and stability.

## Materials and Methods

### Strains and media.

Chlamydomonas reinhardtii wild-type and mutant cells were grown as described [48]. The *stt7* mutant and *stt7* complemented with Stt7-HA were used [21]. The HA-tag consists of six copies of the HA peptide YPYDVPDYA inserted at the C-terminal end of Stt7. In some experiments, the double-tagged FLAG-Stt7-HA was used with FLAG inserted after the Stt7 transit peptide and the HA-tag at the C-terminal end of Stt7. Strains were maintained on Tris-acetate-phosphate (TAP) medium at 25 °C in dim light (10 μmol m^−2^ s^−1^). The *stt7* mutant strain complemented with Stt7-HA was also transformed with the ph6FA1 plasmid [25] in order to obtain a strain expressing Stt7-HA and cytochrome *f* tagged with a His-tag. Homoplasmicity of this strain was checked by PCR.

### Tagging of Stt7.

Stt7-HA consists of six copies of the HA epitope YPYDVPDYA inserted at the C-terminal end of Stt7. The molecular mass of this HA tag is 8.4 kDa and that of Stt7-HA is 85 kDa. Compared with Stt7-HA, FLAG-Stt7-HA contains in addition the FLAG tag RDYKDHDGDYKDHDIDYKDDDDKS with a molecular mass of 3 kDa inserted after the 41 amino acid transit peptide of Stt7.

### Analysis of thylakoid membranes in state 1 and state 2.

Cultures were grown in TAP medium to a density of 2 × 10^6^ cells/ml. Cells were subsequently concentrated 10-fold in HSM medium. State 1 was induced by incubating cells in 10^−5^ M DCMU (3-(3,4-dichlorophenyl)-1,1-dimethylurea) in dim light (10 μmol m^−2^ s^−1^) under strong aeration, and state 2 was obtained by incubating cells under anaerobic conditions in the dark. Cells were harvested at 6,000*g* for 10 min and resuspended in buffer at a density of 10^8^ cells/ml and broken in a French press at 1,200 psi. Thylakoid membranes (0.8 mg/ml) were prepared as described [11] and solubilized with 0.9% n-dodecyl-β-maltoside for 30 min in ice, then 0.5 ml were layered on a sucrose density gradient (0.1–1.3 M sucrose in 5 mM Tricine-NaOH [pH 8.0], 0.05% n-dodecyl-β-maltoside) and centrifuged at 280,000*g* for 16 h in a SW40 Beckman rotor. After centrifugation, the gradient was divided into 18 fractions that were analyzed by SDS/PAGE and immunoblotting. The antibodies used were against HA, FLAG, Cyt*f*, PsaA, D1, CP26, CP29, Lhcbm5, and P-Thr.

### Protein stability measurements during state transitions.

Cultures were grown in TAP medium to a density of 2 × 10^6^ cells/ml. After a 10-fold concentration, cells were incubated in HSM medium under anaerobic conditions in the dark for 2 h. Cells were then maintained under state 2 conditions or cultured under dim light under strong aeration (state 1 conditions). In some cases, cycloheximide (10 μg/ml) or protease inhibitor cocktail (Roche) (2× concentration recommended by the manufacturer) were added prior to the onset of state 1 conditions.

### Fluorescence measurements.

Chlorophyll fluorescence emission spectra were recorded with a Jasco FP-750 spectrofluorimeter using intact cells at a concentration of 10^6^ cells/ml frozen in liquid nitrogen. The excitation light had a wavelength of 435 nm, and emission was detected from 650 to 800 nm. Fv and Fmax measurements at room temperature were performed with a Hansatech PAM fluorimeter.

### Immunoblot analysis of proteins phosphorylated during state transitions.

Proteins from thylakoid membranes isolated from cells in either state 1 or state 2 were separated on a 15% SDS-polyacrylamide 6 M urea gel and transferred to nitrocellulose membranes. The membranes were blocked with bovine serum albumin and incubated with rabbit anti-phosphothreonine antibody (Cell Signaling Technology).

### Immunoprecipitations and pull-down experiments.

Thylakoid membranes (0.8 mg/ml) were solubilized with n-dodecyl-β-maltoside for 30 min in ice, and nonsolubilized material was removed by centrifugation at 12,000*g* for 10 min at 4 °C. Fifty microliters of anti–HA-affinity matrix was added to 0.3 mg of chlorophyll of solubilized membranes and incubated overnight at 4 °C. The beads were washed five times in TBS-BSA (100 mM Tris/HCl [pH 7.5], 150 mM NaCl, 0.05% BSA), and the bound proteins were eluted in 40 μl of 2× SDS loading buffer (100 mM Tris/HCl [pH 6.8], 4% SDS, 0.2% bromophenol blue, 20% glycerol) for 30 min at room temperature. The immunoprecipitated proteins were analyzed by immunoblotting with antibodies against subunits of the photosynthetic complexes.

The full-length Stt7 cDNA was cloned in the pGEX-4T-1 expression vector (Amersham Pharmacia Biotech). Proteins were expressed in Escherichia coli as GST fusion and purified with the GST fusion system kit (Amersham Pharmacia Biotech). Two micrograms of GST fusion protein were immobilized on glutathione Sepharose 4B beads (incubation for 2 h at 4 °C) and mixed with 50 μg of solubilized thylakoid extract in 0.5 ml of PBS. After overnight incubation at 4 °C on a rotary shaker, beads were washed four times with the same buffer, resuspended in 30 μl of SDS gel-loading buffer (100 mM DTT, 2% SDS), and the eluate was fractionated by SDS-PAGE.

### Proteolysis of thylakoid membranes.

Thylakoids were washed three time in 50 mM Hepes (pH 7.4), 0.3 M sucrose [49] without protease inhibitors and resuspended in NH_4_HCO_3_ 25 mM. Samples were sonicated in a waterbath sonicator with 1-min sonication and 30-s cooling five times. Endoproteinase GLU-C (Sigma) was added at indicated concentrations to thylakoid membranes at a chlorophyll concentration of 0.3 mg/ml at room temperature for 15 min. The digestion was arrested by TCA precipitation and samples were resuspended in 1× loading buffer containing 8 M Urea. Further analyses of the samples were performed by SDS-PAGE and immunoblotting.

### Isolation of cytochrome *b*_6_ *f* complex.

All steps were performed at 4 °C with 1 mM AEBSF (Roche) as protease inhibitor in all buffers. After solubilization with 0.9% n-dodecyl-β-maltoside, 640 μg of thylakoid membranes (chlorophyll equivalent) were incubated 2 h with Ni-NTA matrix (Qiagen) in the presence of 200 mM NaCl and 10 mM imidazole. The matrix was washed five times in washing buffer (TBS, 200 mM NaCl, 20 mM imidazole), and the bound proteins were eluted in 2× 1 ml of elution buffer (TBS, 250 mM NaCl, 300 mM imidazole). The eluted proteins were analyzed by immunoblotting with antibodies against subunits of the photosynthetic complexes.

### Split-ubiquitin system.

The split-ubiquitin experiments were performed using the DUALmembrane kit 3 from Dualsystems Biotech (http://www.dualsystems.com). Cub fused to the artificial transcription factor LexA-VP16 was fused to either the N-terminal end of Stt7 (minus the chloroplast signal peptide) or to its C-terminal end (including STE leader sequence) according to the manufacturer's protocol using SfiI restriction sites. These constructs were then tested for their ability to release LexA-VP16 when coexpressed with an integral membrane protein (Alg5) fused at its C-terminal to the N-terminal half of ubiquitin (Nub). If both Cub and Nub are located in the cytoplasm, spontaneous reassociation will occur, and ubiquitin-specific proteases will be recruited, releasing LexA-VP16, which will in turn activate the reporter genes (Ade and His).

## Supporting Information

Figure S1 Determination of Molar Ratio of Stt7 Kinase and the Cytochrome *b*_6_ *f* Complex Upper panels: amounts loaded are indicated in percents (cytochrome *b*_6_ *f* complex) and nanograms per micrograms (recombinant Stt7 and BSA). Proteins were separated by PAGE and stained with Coomassie Blue. The circled bands correspond to the same amount of protein. Lower panels: total proteins from wild-type cells were fractionated by SDS-PAGE and immunoblotted with the indicated antisera. Quantification was performed by comparing the signals with known amounts of recombinant Stt7 protein and Cyt*f* derived from the upper panels. Bands corresponding to the same signal are circled. A molar ratio of 1:20 was estimated by taking into account the dilutions and the molecular weights of Stt7 (80,000) and cytochrome *f* (32,000). The same ratio was obtained using SYPRO Ruby staining (Invitrogen) instead of Coomassie staining for quantification of recombinant Stt7 and purified cytochrome *b*_6_ *f* complex.
(2.96 MB EPS)

Figure S2 PSI, PSII, and the Cytochrome *b*_6_ *f* Complex Accumulate to the Same Levels in Wild Type, Stt7-HA, and *stt7*
Dilution series were used to compare the levels of the indicated proteins by PAGE and immunoblotting. In the case of Stt7, the level of the protein represents 25%–50% of wild-type levels.
(4.22 MB EPS)

Figure S3 Decrease of Stt7 Level under Prolonged State 1 Conditions (A) Cells containing Stt7-HA were first grown for 120 min under state 2 conditions and then shifted to state 1 as in Figure 2C except that the immunoblotting was performed with Stt7 and HA antisera. The band corresponding to Stt7 is marked with an arrow. The band below Stt7 is unspecific and is also seen in the *stt7* mutant. For unknown reasons, the intensity of this band varies from one experiment to another.
(B) Same as (A) except that wild-type cells with untagged Stt7 were used, and the immunoblotting was performed with Stt7 antiserum. Cyt*f* was used as loading control.
(1.15 MB EPS)

Figure S4 Degradation of Stt7 under State 1 Conditions Is Blocked by Cys Protease Inhibitors Cells containing Stt7-HA were cultured under state 2 conditions for 90 min as in Figure 2.
(A and B) Cells were subsequently subjected to state 1 conditions with addition of different protease inhibitors (PIC, ACA, AEBSF, NEM, PMSF, and EDTA), and aliquots were collected after 0, 1, and 2 h and examined by immunoblotting with the indicated antibodies.
(C, D, and E) Cells were subjected to state 1 conditions with increasing concentrations of E64, leupeptin, and NEM. Aliquots were collected after 0, 2, and 4 h and examined by immunoblotting with the indicated antibodies.
I, initial time point after the initial culture was maintained in state 2 for 120 min; ∅︀, no treatment.
(1.67 MB EPS)

Text S1 Bioinformatic Search of Proteases (24 KB DOC)

## Abbreviations

Cub: C-terminal fragment of ubiquitin
Cytf: cytochrome f
Fmax: maximum fluorescence
Fv: variable fluorescence
HA: haemagglutinin
Nub: N-terminal end of ubiquitin
PSI: photosystem I
PSII: photosystem II

## References

[pbio-1000045-b001] Holt NE, Fleming GR, Niyogi KK (2004). Toward an understanding of the mechanism of nonphotochemical quenching in green plants. Biochemistry.

[pbio-1000045-b002] Allen JF (1992). Protein phosphorylation in regulation of photosynthesis. Biochim Biophys Acta.

[pbio-1000045-b003] Rochaix JD (2007). Role of thylakoid protein kinases in photosynthetic acclimation. FEBS Lett.

[pbio-1000045-b004] Wollman FA (2001). State transitions reveal the dynamics and flexibility of the photosynthetic apparatus. EMBO J.

[pbio-1000045-b005] Vener AV, van Kan PJ, Rich PR, Ohad II, Andersson B (1997). Plastoquinol at the quinol oxidation site of reduced cytochrome bf mediates signal transduction between light and protein phosphorylation: Thylakoid protein kinase deactivation by a single-turnover flash. Proc Natl Acad Sci U S A.

[pbio-1000045-b006] Zito F, Finazzi G, Delosme R, Nitschke W, Picot D (1999). The Qo site of cytochrome b6f complexes controls the activation of the LHCII kinase. EMBO J.

[pbio-1000045-b007] Minagawa J, Takahashi Y (2004). Structure, function and assembly of Photosystem II and its light-harvesting proteins. Photosynth Res.

[pbio-1000045-b008] De Vitry C, Wollman FA (1988). Changes in phosphorylation of thylakoid membrane proteins in light-harvesting complex mutants from Chlamydomonas reinhardtii. Biochim, Biophys Acta.

[pbio-1000045-b009] Fleischmann MM, Ravanel S, Delosme R, Olive J, Zito F (1999). Isolation and characterization of photoautotrophic mutants of Chlamydomonas reinhardtii deficient in state transition. J Biol Chem.

[pbio-1000045-b010] Kargul J, Turkina MV, Nield J, Benson S, Vener AV (2005). Light-harvesting complex II protein CP29 binds to photosystem I of Chlamydomonas reinhardtii under State 2 conditions. FEBS J.

[pbio-1000045-b011] Takahashi H, Iwai M, Takahashi Y, Minagawa J (2006). Identification of the mobile light-harvesting complex II polypeptides for state transitions in Chlamydomonas reinhardtii. Proc Natl Acad Sci U S A.

[pbio-1000045-b012] Lunde C, Jensen PE, Haldrup A, Knoetzel J, Scheller HV (2000). The PSI-H subunit of photosystem I is essential for state transitions in plant photosynthesis. Nature.

[pbio-1000045-b013] Bulté L, Gans P, Rebeille F, Wollman FA (1990). ATP control on state transitions in Chlamydomonas. Biochim Biophys Acta.

[pbio-1000045-b014] Finazzi G, Barbagallo RP, Bergo E, Barbato R, Forti G (2001). Photoinhibition of Chlamydomonas reinhardtii in State 1 and State 2: damages to the photosynthetic apparatus under linear and cyclic electron flow. J Biol Chem.

[pbio-1000045-b015] Finazzi G, Rappaport F, Furia A, Fleischmann M, Rochaix JD (2002). Involvement of state transitions in the switch between linear and cyclic electron flow in Chlamydomonas reinhardtii. EMBO Rep.

[pbio-1000045-b016] Hou CX, Rintamaki E, Aro EM (2003). Ascorbate-mediated LHCII protein phosphorylation–LHCII kinase regulation in light and in darkness. Biochemistry.

[pbio-1000045-b017] Tikkanen M, Piippo M, Suorsa M, Sirpio S, Mulo P (2006). State transitions revisited-a buffering system for dynamic low light acclimation of Arabidopsis. Plant Mol Biol.

[pbio-1000045-b018] Delosme R, Olive J, Wollman FA (1996). Changes in light energy distribution upon state transitions: an in vivo photoacoustic study of the wild type and photosynthesis mutants from Chlamydomonas reinhardtii. Biochim Biophys Acta.

[pbio-1000045-b019] Chuartzman SG, Nevo R, Shimoni E, Charuvi D, Kiss V (2008). Thylakoid membrane remodeling during state transitions in Arabidopsis. Plant Cell.

[pbio-1000045-b020] Kruse O, Nixon PJ, Schmid GH, Mullineaux CW (1999). Isolation of state transition mutants of Chlamydomonas reinhardtii by fluorescence video imaging. Photosynthesis Res.

[pbio-1000045-b021] Depège N, Bellafiore S, Rochaix JD (2003). Role of chloroplast protein kinase Stt7 in LHCII phosphorylation and state transition in Chlamydomonas. Science.

[pbio-1000045-b022] Bellafiore S, Barneche F, Peltier G, Rochaix JD (2005). State transitions and light adaptation require chloroplast thylakoid protein kinase STN7. Nature.

[pbio-1000045-b023] Rintamaki E, Martinsuo P, Pursiheimo S, Aro EM (2000). Cooperative regulation of light-harvesting complex II phosphorylation via the plastoquinol and ferredoxin-thioredoxin system in chloroplasts. Proc Natl Acad Sci U S A.

[pbio-1000045-b024] Martinsuo P, Pursiheimo S, Aro EM, Rintamäki E (2003). Dithiol oxidant and disulfide reductant dynamically regulate the phosphorylation of light-harvesting complex II proteins in thylakoid membranes. Plant Physiol.

[pbio-1000045-b025] Stroebel D, Choquet Y, Popot JL, Picot D (2003). An atypical haem in the cytochrome b(6)f complex. Nature.

[pbio-1000045-b026] Johnsson N, Varshavsky A (1994). Split ubiquitin as a sensor of protein interactions in vivo. Proc Natl Acad Sci U S A.

[pbio-1000045-b027] Subramanyam R, Jolley C, Brune DC, Fromme P, Webber AN (2006). Characterization of a novel Photosystem I-LHCI supercomplex isolated from Chlamydomonas reinhardtii under anaerobic (State II) conditions. FEBS Lett.

[pbio-1000045-b028] Adam Z, Clarke AK (2002). Cutting edge of chloroplast proteolysis. Trends Plant Sci.

[pbio-1000045-b029] Helm M, Luck C, Prestele J, Hierl G, Huesgen PF (2007). Dual specificities of the glyoxysomal/peroxisomal processing protease Deg15 in higher plants. Proc Natl Acad Sci U S A.

[pbio-1000045-b030] Bonardi V, Pesaresi P, Becker T, Schleiff E, Wagner R (2005). Photosystem II core phosphorylation and photosynthetic acclimation require two different protein kinases. Nature.

[pbio-1000045-b031] Rintamaki E, Salonen M, Suoranta UM, Carlberg I, Andersson B (1997). Phosphorylation of light-harvesting complex II and photosystem II core proteins shows different irradiance-dependent regulation in vivo. Application of phosphothreonine antibodies to analysis of thylakoid phosphoproteins. J Biol Chem.

[pbio-1000045-b032] Wollman FA, Lemaire C (1988). Studies on kinase-controlled state transitions in photosystem II and b6f mutants from Chlamydomonas reinhardtii which lack quinone-binding proteins. Biochim Biophys Acta.

[pbio-1000045-b033] Hamel P, Olive J, Pierre Y, Wollman FA, de Vitry C (2000). A new subunit of cytochrome b6f complex undergoes reversible phosphorylation upon state transition. J Biol Chem.

[pbio-1000045-b034] Breyton C (2000). Conformational changes in the cytochrome b6f complex induced by inhibitor binding. J Biol Chem.

[pbio-1000045-b035] Zhang Z, Huang L, Shulmeister VM, Chi YI, Kim KK (1998). Electron transfer by domain movement in cytochrome bc1. Nature.

[pbio-1000045-b036] de Lacroix de Lavalette A, Finazzi G, Zito F (2008). b6f-Associated chlorophyll: structural and dynamic contribution to the different cytochrome functions. *Biochemistry*.

[pbio-1000045-b037] Finazzi G, Zito F, Barbagallo RP, Wollman FA (2001). Contrasted effects of inhibitors of cytochrome b6f complex on state transitions in Chlamydomonas reinhardtii: the role of Qo site occupancy in LHCII kinase activation. J Biol Chem.

[pbio-1000045-b038] Gal A, Zer H, Ohad I (1997). Redox-cntrolled thylakoid protein phosphorylation. News and views. Plant Physiol.

[pbio-1000045-b039] Dekker JP, Boekema EJ (2005). Supramolecular organization of thylakoid membrane proteins in green plants. Biochim Biophys Acta.

[pbio-1000045-b040] Dainese P, Bassi R (1991). Subunit stoichiometry of the chloroplast photosystem II antenna system and aggregation state of the component chlorophyll a/b binding proteins. J Biol Chem.

[pbio-1000045-b041] Harrer R, Bassi R, Testi MG, Schafer C (1998). Nearest-neighbor analysis of a photosystem II complex from Marchantia polymorpha L. (liverwort), which contains reaction center and antenna proteins. Eur J Biochem.

[pbio-1000045-b042] Turkina MV, Kargul J, Blanco-Rivero A, Villarejo A, Barber J (2006). Environmentally modulated phosphoproteome of photosynthetic membranes in the green alga Chlamydomonas reinhardtii. Mol Cell Proteomics.

[pbio-1000045-b043] Bassi R, Marquardt J, Lavergne J (1995). Biochemical and functional properties of photosystem II in agranal membranes from maize mesophyll and bundle sheath chloroplasts. Eur J Biochem.

[pbio-1000045-b044] Tikkanen M, Nurmi M, Suorsa M, Danielsson R, Mamedov F (2008). Phosphorylation-dependent regulation of excitation energy distribution between the two photosystems in higher plants. Biochim Biophys Acta.

[pbio-1000045-b045] Page ML, Hamel PP, Gabilly ST, Zegzouti H, Perea JV (2004). A homolog of prokaryotic thiol disulfide transporter CcdA is required for the assembly of the cytochrome b6f complex in Arabidopsis chloroplasts. J Biol Chem.

[pbio-1000045-b046] Lennartz K, Plucken H, Seidler A, Westhoff P, Bechtold N (2001). HCF164 encodes a thioredoxin-like protein involved in the biogenesis of the cytochrome b(6)f complex in Arabidopsis. Plant Cell.

[pbio-1000045-b047] Motohashi K, Hisabori T (2006). HCF164 receives reducing equivalents from stromal thioredoxin across the thylakoid membrane and mediates reduction of target proteins in the thylakoid lumen. J Biol Chem.

[pbio-1000045-b048] Harris EH (1989). The Chlamydomonas sourcebook : a comprehensive guide to biology and laboratory use.

[pbio-1000045-b049] Cline K, Werner-Washburne M, Andrews J, Keegstra K (1984). Thermolysin is a suitable protease for probing the surface of intact pea chloroplasts. Plant Physiol.

