# Supplementary material for: Analysis of the Chloroplast Protein Kinase Stt7 during State Transitions
Source: PLoS Biol. 2009 Mar 3;7(3):e1000045. doi: 10.1371/journal.pbio.1000045 (PMC2650728; doi:10.1371/journal.pbio.1000045)
Supplement: Supplementary file 1 [file pbio.1000045.sd001.doc]

**Bioinformatic search of proteases**

Cys proteases of *A. thaliana* were searched using the Interpro signature IPR000169 (<http://www.ebi.ac.uk/interpro/>). This search of the *A. thaliana* complete proteome in UniProt (<http://www.uniprot.org/>) yielded 53 proteins of which 10 could be assigned to the chloroplast with SUBA (<http://www.plantenergy.uwa.edu.au/applications/suba2/>): At1g02305, At1g09850, At1g20850, At1g29090, At2g21430, At2g27420, At3g19400, At3g45310, At4g39090, At5g45890. However none of these proteins could be assigned to plastids according to Target P.
